# Association between Molar Incisor Hypomineralization in Schoolchildren and Both Prenatal and Postnatal Factors: A Population-Based Study

**DOI:** 10.1371/journal.pone.0156332

**Published:** 2016-06-09

**Authors:** Luciana Fonseca Pádua Gonçalves Tourino, Patrícia Corrêa-Faria, Raquel Conceição Ferreira, Cristiane Baccin Bendo, Patrícia Maria Zarzar, Miriam Pimenta Vale

**Affiliations:** 1 Department of Pediatric Dentistry, Centro Universitário de Lavras, Lavras, Minas Gerais, Brazil; 2 Department of Pediatric Dentistry and Orthodontics, School of Dentistry, Universidade Federal de Minas Gerais, Belo Horizonte, Minas Gerais, Brazil; 3 Department of Public Oral Health, School of Dentistry, Universidade Federal de Minas Gerais, Belo Horizonte, Minas Gerais, Brazil; Université de Poitiers, FRANCE

## Abstract

**Background:**

Although studies throughout the world have investigated potential factors involved in the occurrence of molar incisor hypomineralization (MIH), the findings are varied and inconclusive.

**Objective:**

The aim of the present study was to evaluate the prevalence of MIH and identify associated prenatal, perinatal and postnatal factors among Brazilian schoolchildren aged 8 and 9 years.

**Methods:**

A cross-sectional study was conducted with a randomly selected population-based sample of 1181 schoolchildren. Information on demographic and socioeconomic characteristics as well as prenatal, perinatal and postnatal aspects was obtained through questionnaires. The clinical examination included the investigation of MIH based on the criteria of the European Academy of Paediatric Dentistry. Dental caries in the permanent dentition and developmental defects of enamel (DDE) on the primary second molars were also recorded. Data analysis involved descriptive statistics, bivariate tests and Poisson regression with robust variance.

**Results:**

The prevalence of MIH was 20.4%. MIH was more frequent among children with dental caries in the permanent dentition (PR: 2.67; 95% CI: 1.98–3.61), those with DDE on the primary second molars (PR: 2.54; 95% CI: 1.87–3.45) and those who experienced asthma/bronchitis in the first four years of life (PR: 1.93; 95% CI: 1.45–2.56).

**Conclusions:**

The prevalence of MIH was high and was associated with dental caries, the presence of DDE on primary second molars and the experience of asthma/bronchitis in early life. These findings could be useful in the identification of children in need of shorter recall intervals to prevent the consequences of MIH, such as enamel breakdown dental caries.

## Introduction

Molar incisor hypomineralization (MIH) is characterized by asymmetric opacities of a systemic origin that affect one to four permanent first molars and can affect the permanent incisors [1]. Affected teeth exhibit well-defined, white, cream-colored, yellow or brown opacities on the enamel, varying in extent and severity. In more severe cases, the enamel of the molars undergoes post-eruptive breakdown, which facilitates the development of dental caries [2] and causes extreme hypersensitivity that often results in severe discomfort [3]. Thus, such patients have a greater need for clinical interventions [4], and dentists face considerable challenges providing restorative treatment for hypomineralized molars [5]. Additionally, patients face aesthetic problems when the incisors are affected [2].

The prevalence rate of MIH varies in different populations, due to age of the subjects, methodological differences and criteria used in the diagnosis of this alteration. According to epidemiological studies, the lowest prevalence (2.5%) of MIH was observed among Chinese children [6], while another study conducted in Brazil showed the prevalence rate to be 40.2% [7]. However, two other studies performed later in Brazil revealed a prevalence rate of 12.3% [8] and 19.8% [4], similar to recent studies conducted in Germany [9], Iraq [10] and Spain [11], which found a prevalence rate of 10.1–21.8%. The variations between the Brazilian studies are probably due to methodological differences and the criteria used in the diagnosis of this alteration. In the first study [7] the extension in a lesion was not considered as a criterion for diagnosis and a convenience sample was used.

The etiology of MIH is related to complications during the mineralization period of the permanent first molars and incisors. The mineralization of these teeth begins at the end of the gestation period and is completed throughout the first four years of life. Thus, abnormalities in this period are related to the occurrence of MIH [12]. Such abnormalities include premature birth and low birth weight [10,13,14], hypocalcaemia [10] and diseases such as chicken pox [15] and asthma [16] as well as frequent episodes of fever in early childhood [10]. Although many studies around the world have investigated potential factors involved in the occurrence of MIH, the findings have been varied and inconclusive [10, 12–18]. According to reviews on MIH, there is insufficient evidence regarding the factors associated with MIH etiology. Evidence of the role of the neonatal aspects and childhood diseases are still weak and compromise the establishment of a causal relationship to MIH. The published research fails in relation to the study design and lack of standardization of the criteria for diagnosing MIH [19,20]. Moreover, many possible factors associated with MIH are highly correlated, and the majority of studies fail to control for confounding variables in the statistical analyses [12,14–16,18].

Knowledge on factors associated with the etiology of MIH can contribute to the identification of children who are more prone to this condition as well as the establishment of preventive measures and specific treatment. However, few population-based studies have addressed prenatal, perinatal and postnatal factors associated with the occurrence of MIH in Brazilian children [21]. Thus, the aim of the present study was to describe the prevalence of MIH among Brazilian children aged 8 and 9 years and investigate potential associated prenatal, perinatal and postnatal factors.

## Material and Methods

### Ethics statement

This study was approved by the Human Research Ethics Committee of the Federal University of Minas Gerais, Brazil (reg. n° 10659812.0.0000.5149). Written statements of consent were read and signed by mothers and children prior to their participation in the study.

### Calibration of the examiner

Prior to the clinical examination, the single examiner (LFPGT) was trained by an experienced pediatric dentist (gold standard) to identify MIH in the permanent dentition and developmental defects of enamel (DDE) in the primary dentition. The training and calibration exercise consisted of three stages. The theoretical step involved a discussion of the criteria for the diagnosis of the enamel defects investigated [1, 22]. The second stage involved the analysis of photographs on two occasions with a two-week interval between sessions. Clinical photographs of 183 primary and permanent teeth with enamel defects covering all degrees of MIH, fluorosis, hypoplasia and amelogenesis were evaluated, along with sound teeth. Data analysis involved the calculation of Kappa coefficients. Inter-examiner (between examiner and gold standard) and intra-examiner agreement was tested (Kappa = 0.78 to 0.97 and 0.84 to 1.00 for DDE and MIH, respectively).

Clinical examinations were performed on 30 children aged 8 and 9 years on two occasions with a two-week interval between examinations. The intra-examiner and inter-examiner Kappa coefficients were 0.85 to 1.00 and 0.80 for MIH, respectively, and 0.86 to 1.00 and 0.97 for DDE, respectively. Moreover, the single examiner was trained and calibrated for the diagnosis of dental caries in the permanent dentition using the criteria of the World Health Organization (WHO) [23]. For such, intra-examiner agreement was determined (Kappa = 0.96).

### Questionnaire reliability

A self-administered questionnaire adapted from Jälevik et al. [18] addressed questions regarding prenatal, perinatal and health characteristics of the child in the first four years of life was used in the study. As the questionnaire was originally drafted in English, it was submitted to translation and cross-cultural adaptation based on the protocol proposed by Guillermin et al. [24]. The Portuguese version of the questionnaire was evaluated by a review board composed of three specialists in pediatric dentistry, with experience in health questionnaire/instrument validation, who were fluent in both Portuguese and English (Drs. MP Vale, PM Zarzar, SM Paiva). The review board identified possible difficulties in understanding the questionnaire and made the necessary modifications. Moreover, questions derived from the available literature on supposed etiological factors of MIH, such as asthma [16] and high fever [10] in the first four years of the child’s life, were added to the questionnaire. The questionnaire was then self-administered by 30 mothers who accompanied their children during routine visits to the dental office. The same mothers answered the questionnaire a second time after a 15-day interval (first test-retest). Test-retest reliability was determined using the Kappa coefficient. Based on the findings, the review board made changes to some of the questions did not achieve good reproducibility, i.e., Kappa value was <0.60 [25] and to those that the mothers had difficulty answering. The questions about the occurrence of disease and the use of medicine in the first year and in the second, third, and fourth year of life were grouped together in the first four years of life to minimize recall bias. After these changes, a second test-retest was performed in the same way as the first, but with 30 mothers who did not participate in the first test-retest. After an analysis of the findings, only one question was changed by the review board and the final version of the questionnaire (S1 File) was tested in a pilot study.

### Pilot study

The study methods, dental examinations, administration of the questionnaire and preparation of the examiner were tested in a pilot study with a convenience sample of 67 children and their mothers who were not included in the main study. Among these 67 mothers, 49 completed the questionnaire on two occasions with a two-week interval between sessions. Test-retest reliability of the questionnaire was determined using the Kappa coefficient. Questions addressing the use of antibiotics (Kappa = 0.56) and the occurrence of influenza and colds in the first four years of life (Kappa = 0.55) achieved Kappa coefficients below 0.60. All other questions achieved Kappa coefficients ranging from 0.62 to 1.00. Following the analysis of the review board, there was no need for further changes to the questionnaire. Moreover, no changes to the assessment tools or proposed methodology were deemed necessary.

### Study area and design

A population-based, cross-sectional study was conducted from March to September 2014 with a representative sample of schoolchildren aged 8 and 9 years in the city of Lavras, which is located in the state of Minas Gerais (southeast Brazil). This city has 92,171 inhabitants and the Human Development Index is 0.819 [26]. The natural level of fluoride in the rural community water is below 0.1 ppm/F and the ion concentration in the urban community water after fluoridation is 0.7 ppm/F.

### Sampling procedure

Participants were selected from children attending the 3^rd^ and 4^th^ grades at 10 private and 24 public primary schools in both urban and rural areas of Lavras. The method used to calculate the sample size was the comparison of two proportions, i.e., individuals with MIH and without MIH, with correction for a finite population. A pilot study was conducted to determine the frequency of MIH between exposed and unexposed individuals to the key independent variables (birth weight, neonatal medical care, breastfeeding duration, illnesses, pneumonia, asthma/bronchitis, use of medication, history of hospitalization and fever higher than 38.5°C in the first four years of life). The sample size was calculated for each of the mentioned independent variables to give a power of 80% and a 5% significance level; the largest value obtained was adopted. Considering the possible losses, 20% was added to the sample size, resulting in a required minimum of 1389 children.

The selection of children was randomized and stratified by type of school (public or private) to ensure the representativeness of the sample. The Epi Info program (version 6.0) was used for the randomization procedure. In cases of refusals to participate, incomplete questionnaires and absence from school on the day of the exam, a replacement randomization procedure was performed. For such, a child from the same classroom was randomly selected.

The inclusion criteria were children aged between 8 and 9 years 11 months who were lifelong residents of the city of Lavras and had all permanent incisors and first molars fully erupted. Children with syndromes connected to enamel malformations, those with amelogenesis imperfecta and children wearing fixed orthodontic appliances were excluded.

### Data Collection

The children were examined in the school setting following the WHO guidelines [23] under natural light outdoors by a single calibrated examiner and a trained assistant, who recorded the observations. Prior to the exam, the teeth were brushed by the children under the supervision of the examiner. The teeth were dried with sterile gauze and the clinical examination was performed with disposable tongue depressors, standard mouth mirrors and probes. During the exam, the child remained seated in front of the examiner.

The criteria proposed by the European Academy of Paediatric Dentistry (EAPD) [1] were used for the diagnosis of MIH, which include the presence of demarcated opacities, post-eruptive enamel breakdown, atypical restorations and extraction due to MIH in at least one first permanent molar. Demarcated opacities with a diameter of < 1 mm were not considered in the analysis [23].

The primary second molars were also examined for three types of enamel defects (demarcated opacities, diffuse opacities and hypoplasia) and were classified based on the criteria of the modified Index of Developmental Defects of Enamel [22]. Teeth with up to two thirds of the crown restored and those with deep caries or fractures were not considered and received a classification of “not recorded”. DDE was recorded if the child had at least one primary second molar with an enamel defect.

Dental caries experience was recorded using the WHO criteria for diagnosis of decayed, missing and filled teeth (DMFT Index) [23]. The examination for dental caries included all permanent teeth.

The mentioned self-administered questionnaire (S1 File) addressing questions regarding prenatal, perinatal and health characteristics of the child in the first four years of life was sent to the mothers. Another questionnaire addressing socioeconomic indicators (monthly household income and mothers' schooling), child’s sex and age was also sent to the mothers.

Independent variables were grouped according to socioeconomic and demographic characteristics, chronological phase of exposure to possible associated factors (prenatal, perinatal and postnatal characteristics) and oral characteristics (Table 1).

**Table 1:** Frequency distribution of MIH and crude prevalence ratio according to socioeconomic, demographic, prenatal, perinatal, postnatal and oral health characteristics (n = 1181).

	Absence of MIH n (%)	Presence of MIH n (%)	Crude PR (95% CI)	p-value^1^
**Socioeconomic and demographic characteristics**				
Child’s sex				
Male	466 (80.1%)	116 (19.9%)	1	
Female	474 (79.1%)	125 (20.9%)	1.04 (0.81–1.35)	0.722
Mother’s schooling^a^				
>8 years	436 (77.6%)	126 (22.4%)	1	
≤8 years	448 (82.9%)	98 (17.9%)	0.80 (0.61–1.04)	0.099
Monthly household income^b^				
Up to minimum wage	159 (79.9%)	40 (20.1%)	1	
>1 to 2 times minimum wage	274 (79.4%)	71 (20.6%)	1.02 (0.69–1.51)	0.905
>2 to 3 times minimum wage	210 (79.2%)	55 (20.8%)	1.03 (0.69–1.55)	0.878
>3 times minimum wage	175 (79.5%)	45 (20.5%)	1.02 (0.66–1.56)	0.936
**Prenatal characteristics**				
Pre-eclampsia^c^				
No	903 (79.9%)	227 (20.1%)	1	
Yes	33 (71.7%)	13 (28.3%)	1.41 (0.80–2.46)	0.231
Use of medication to avoid premature birth^c^				
No	878 (80.3%)	215 (19.7%)	1	
Yes	49 (70.0%)	21 (30.0%)	1.53 (0.97–2.39)	0.065
Use of paracetamol^c^				
No	868 (80.4%)	212 (19.6%)	1	
Yes	59 (71.1%)	24 (28.9%)	1.47 (0.97–2.25)	0.072
**Perinatal characteristics**				
Premature birth^c^				
No	865 (80.4%)	211 (19.6%)	1	
Yes	72 (71.3%)	29 (28.7%)	1.46 (0.99–2.16)	0.054
Oxygenation without intubation^c^				
No	927 (79.5%)	229 (19.9%)	1	
Yes	13 (86.7%)	12 (42.9%)	2.16 (1.21–3.86)	**0.009**
**Postnatal characteristics**				
Breastfeeding duration^c^				
<6 month	372 (77.0%)	111 (23.0%)	1	
≥6 months	568 (81.4%)	130 (18.6%)	0.81 (0.60–1.04)	0.068
Illnesses up to 4 years of age^c^				
No	278 (85.3%)	48 (14.7%)	1	
Yes	660 (77.4%)	193 (22.6%)	1.54 (1.12–2.11)	**0.008**
Pneumonia^c^				
No	824 (81.0%)	193 (19.0%)	1	
Yes	114 (70.4%)	48 (29.6%)	1.56 (1.14–2.14)	**0.006**
Asthma/bronchitis^c^				
No	665 (84.3%)	124 (15.7%)	1	
Yes	271 (69.8%)	117 (30.2%)	1.92 (1.49–2.47)	**<0.001**
Use of antibiotics^c^				
No	425 (83.0%)	87 (17.0%)	1	
Yes	514 (77.1%)	153 (22.9%)	1.35 (1.04–1.76)	**0.025**
Use of analgesics^c^				
No	459 (82.1%)	100 (17.9%)	1	
Yes	480 (77.4%)	140 (22.6%)	1.26 (0.98–1.63)	0.075
Use of medication for asthma^c^				
No	663 (84.0%)	126 (16.0%)	1	
Yes	274 (70.4%)	115 (29.6%)	1.85 (1.44–2.38)	**<0.001**
History of hospitalization				
No	701 (81.42%)	160 (18.6%)	1	
Yes	236 (74.9%)	79 (25.1%)	1.35 (1.03–1.77)	**0.029**
Fever higher than 38.5°C				
No	334 (82.1%)	73 (17.9%)	1	
Yes	592 (78.5%)	162 (21.5%)	1.20 (0.91–1.58)	0.200
**Oral characteristics**				
Caries experience in permanent dentition (DMFT≥1)				
No	746 (87.8%)	104 (12.2%)	1	
Yes	194 (58.6%)	137 (41.4%)	3.38 (2.62–4.36)	**<0.001**
DDE on primary 2^nd^ molars^d^				
No	648 (89.4%)	77 (10.6%)	1	
Yes	244 (61.3%)	154 (38.7%)	3.64 (2.77–4.79)	**<0.001**

PR, prevalence ratio; CI, confidence interval; statistically significant differences in bold type (p < 0.05).

^1^Chi-square test (p < 0.05).

^a^Mother’s schooling: data missing from 73 subjects (6.18%).

^b^Household income: data missing from 152 subjects (12.87%).

^c^Number differs from total subjects due to fact that some mothers marked "unknown" option on questionnaire item.

^d^DDE on primary second molars: data missing from 58 subjects due to fact that children received classification of “not recorded” or “missing” for all such teeth.

## Data analysis

Statistical analysis was performed using the STATA program version 12.0 (Stata Corporation, College Station, USA). The chi-square test was used to determine associations between the dependent variable (MIH) and the independent variables (p < 0.05). Poisson regression with robust variance was performed for the analysis of factors associated with MIH. The magnitude of each association was assessed using crude and adjusted prevalence ratios (PRs), respective 95% confidence intervals (CIs) and p-values (Wald test). Univariate regressions were first performed to determine the effect measure of each of the independent variables in the presence of MIH. For the adjustment of the final multiple model, variables were introduced into the model based on their statistical significance (*P* ≤ 0.20) and/or clinical epidemiological importance. In the adjusted final model, variables associated with MIH (*P* < 0.05) and those that improved the fit of the model were considered significantly associated with the outcome and remained in the model. Prevalence ratios (PRs) and 95% confidence intervals (CIs) were calculated.

## Results

A total of 1181 children participated in the study. The response rate was 85%, and the main reason for refusal was that the children forgetting to ask their mother to sign the statement of informed consent. Two hundred and forty one chidren (20.4%) were affected with MIH. The male-to-female ratio was 1:1.03. Nearly half of the children (47.1%) lived in families with a household income greater than two times the Brazilian monthly minimum wage, which corresponded to nearly US$ 252 at the time of the data collection. Table 1 displays the results of the associations between MIH and demographic, socioeconomic, prenatal, perinatal, postnatal and oral health variables in the univariate regression models. MIH was significantly associated with oxygenation without intubation at birth (p = 0.009) as well as the occurrence of illnesses (p = 0.008), pneumonia (p = 0.006), asthma and/or bronchitis (p < 0.001), hospitalization (p = 0.029) and the use of antibiotics (p = 0.025) in the first four years of life. Caries experience in the permanent dentition (p < 0.001) and DDE on primary second molars (p < 0.001) were associated with MIH. No significant associations were found between MIH and household income or mother’s schooling.

After adjusting the model for child’s sex, household income, mother’s schooling and oxygenation without intubation, the following variables remained associated with MIH: caries in the permanent dentition (PR: 2.67; 95%CI: 1.98 to 3.61), DDE on the primary second molars (PR: 2.54; 95%CI: 1.87 to 3.45) and the experience of asthma and/or bronchitis in the first four years of age (PR: 1.93; 95%CI: 1.45 to 2.56) (Table 2).

**Table 2:** Prevalence ratio and confidence interval for associations between MIH and independent variables (n = 1181).

	Adjusted PR^1^	95%CI	p-value
Caries in permanent dentition			
No	1	-	
Yes	2.67	1.98–3.61	<0.001
DDE on primary 2^nd^ molars			
No	1	-	
Yes	2.54	1.87–3.45	<0.001
Asthma and/or bronchitis up to 4 years of age			
No	1	-	
Yes	1.93	1.45–2.56	<0.001

Model adjusted for sex, household income, mother’s schooling and oxygenation without intubation.

^1^Poisson regression.

## Discussion

In this study, the prevalence of MIH was 20.4% among Brazilian schoolchildren aged 8 and 9 years. This prevalence rate is comparable to rates reported in recent studies conducted in Iraq [10] and Spain [11], but higher than rates reported in studies involving Chinese and German children [6, 9]. These variations can be explained by ethnic differences and difference in the age of the children. In Brazil, the first study on MIH conducted with children aged 7 to 13 years reported a prevalence rate of 40.2% [7]. Two later studies from Brazil [4, 8] were conducted with children aged 6 to 12 years and have reported prevalence rates of 19.8% and 12.3%, respectively. In the present study, the choice of age group (8 and 9 years), when all molars and incisors are generally erupted, is considered appropriate for the evaluation of MIH [27] because this age group diminishes the risk of enamel defects being masked by dental caries or restorations. To ensure consistency in the evaluation of MIH, the examinations were performed by a single examiner who had undergone a training and calibration exercise. Moreover, the children randomly selected, with the inclusion of both types of schools (public and private) in both urban and rural areas, which ensured the representativeness of the sample.

No statistically significant associations were found between the occurrence of MIH and mother’s schooling or household income (socioeconomic indicators), which is in agreement with data described in previous studies [8,13,28]. According to a Finnish birth cohort study, mother’s socioeconomic status had no significant direct impact on the prevalence of asthma [29], which was associated with the occurrence of MIH in the present study. The finding that asthma and/or bronchitis in early childhood are associated with the occurrence of MIH in the permanent dentition is in agreement with data described in previous studies [16–18]. A case-control study involving 136 Brazilian children and adolescents (68 asthma patients and 68 controls) aged 5 to 15 years demonstrated that asthma patients had a greater chance of having enamel defects than those without asthma and the enamel defects were not associated with the onset of treatment or the frequency of medication use [16]. However another investigation reported no association between MIH and asthma [15], which may be explained by the smaller sample size in the study. The process of enamel formation is genetically controlled, but sensitive to environmental disturbances [30]. A shortage in the supply of calcium phosphate and oxygen [31] as well as sustained fever [32] can disrupt the normal process of amelogenesis. Thus, one may put forth the hypothesis that previous episodes of oxygen deprivation could have occurred in children with asthma and/or bronchitis, which could have had detrimental effects on amelogenesis. Thus, enamel defects may be associated to the condition itself rather than its treatment [16].

The use of medication to avoid a premature birth, the use of paracetamol/acetaminophen during pregnancy, premature birth, and oxygenation without intubation at birth were not associated with MIH in the multivariate analysis. These finding differ from data reported in previous studies, which found that prenatal [10] and perinatal health conditions [10, 17] were significantly more likely to be associated with the development of MIH. Such divergences may have occurred due to fact that premature infants may be more likely to require intubation and have asthma than full-term infants [33].

In the present study, MIH in children with dental caries experience in the permanent dentition was more than twice as frequent in comparison to those without dental caries, which is in agreement with findings described in previous studies [4,9,17]. This may at least partially be explained by the fact that hypomineralized molars can undergo post-eruptive enamel breakdown, which can result in atypical cavities, requiring extensive restorative treatment or rendering the tooth susceptible to caries. Moreover, such children may be reluctant to perform effective oral hygiene due to hypersensitivity [2].

An association was found between MIH in the permanent dentition and DDE on the primary second molars, which is consistent with data described in previous studies [9,28]. In a study involving 809 Iraqi children aged 7 to 9 years, 53 subjects (6.6%) were diagnosed with at least one hypomineralized primary second molar. Among these 53 subjects, 21 (39.6%) also had hypomineralized permanent first molars, although the association did not achieve statistical significance, most likely due the small sample size used for demarcated lesions. This Iraqi study used criteria adapted from the EAPD for diagnosing MIH in the primary dentition [34].

The present findings should be evaluated with caution. The cross-sectional design only allows the determination of factors associated with MIH and does not permit the establishment of causality. Furthermore, this study was limited by the retrospective collection of exposure information. Although mothers pay considerable attention to their children in the first years of life, it can be difficult for them to remember health problems that affected their children eight and nine years earlier. Attempts were made to minimize recall bias using several methods: the mothers were unaware of the diagnosis of MIH when they completed the questionnaire; the questionnaire had been cross-culturally adapted and the test-retest reliability had been evaluated prior to the study; and the examiner was unaware of the medical history of the children at the time of the dental examination.

The association between MIH and asthma experience in the first four years of life underscores the importance of a multidisciplinary approach. Pediatricians should be aware that children with asthma are more prone to MIH and alert parents to the need for greater oral health care for such children. Pediatric dentists should also have knowledge regarding the general health of their patients to prevent and control possible future threats to the dentition of children with asthma. Moreover, the identification of the association between MIH and enamel defects on primary second molars could contribute to the control of MIH. From the diagnosis of enamel defects on primary second molars, dentists could establish smaller recall intervals. This approach would enable treatment in the early stages of the disease, thereby minimizing future sensitivity and discomfort, assisting in the establishment of oral hygiene measures and controlling for other factors associated with dental caries, as this study has shown that MIH is associated with a higher prevalence rate of dental caries. Nonetheless, prospective studies are needed to assist in clarifying risk factors for the occurrence of MIH, which, in turn, could provide better estimates of the associations.

## Conclusions

The prevalence of MIH was high in the present sample. The occurrence of MIH was more frequent in children with caries experience in the permanent dentition, those with enamel defects on primary second molars and those who had asthma experience in the first four years of life, even after controlling for potential confounding factors. Smaller recall intervals should be established for children with enamel defects on primary second molars and asthma experience in the first four years of life to prevent the consequences of MIH, such enamel breakdown and dental caries.

## Supporting Information

S1 File English version of the questionnaire. (PDF)

S2 File Portuguese version of the questionnaire. (PDF)

S3 File Dataset for the study. (RAR)
