# Supplementary material for: Association between Molar Incisor Hypomineralization in Schoolchildren and Both Prenatal and Postnatal Factors: A Population-Based Study
Source: PLoS One. 2016 Jun 9;11(6):e0156332. doi: 10.1371/journal.pone.0156332 (PMC4900662; doi:10.1371/journal.pone.0156332)
Supplement: S1 File — (PDF) [file pone.0156332.s001.pdf]

QUESTIONNAIRE FOR MOTHERS RELATED TO THE STUDY OF THE FIRST PERMANENT MOLARS IN CHILDREN

Thank you for participating in our study!

Your participation will help us obtain a better understanding of how some teeth come in malformed in some children. Some questions are on your child's health from birth to four years of age and others are on the mother's health during pregnancy. Please, think carefully and try to answer the best way you can.

Child's name:…………………………………………………...……………………..
Mother's name:…………………………………...........…………………………...........

1. How much did your child weigh when he/she was born?
☐ Less than 1.5 kg
☐ From 1.5 kg to 2.49 kg
☐ More than 2.5 kg
☐ I don't know

2. Was your child born premature?
☐ Yes
☐ No
☐ I don't know
If you answered yes, he/she was born after how many weeks?
☐ Less than 28 weeks
☐ From 28 to less than 32 weeks
☐ From 32 to less than 37 weeks
☐ I don't know

<u>3. During the last two months of pregnancy, did you have:</u>
Proteinuria (loss of protein through the urine)? ☐ Yes ☐ No ☐ I don't know
Glucosuria (loss of glucose/blood sugar through the urine)? ☐ Yes ☐ No ☐ I don't know
High blood pressure? ☐ Yes ☐ No ☐ I don't know
Pre-eclampsia? ☐ Yes ☐ No ☐ I don't know
Other serious conditions? ☐ Yes ☐ No ☐ I don't know
If you answered yes, what conditions did you have?...
....

4. During the last four months of pregnancy, did you take any medication?
☐ Yes
☐ No
☐ I don't know
If you answered yes, mark an X next to the medication taken:
☐ Medication for high blood pressure (ex.: Aldomet, etc)
☐ Medication to secure the baby (ex.: Inhibin, etc)
☐ Paracetamol
☐ Other(s). Which?...……………………………………..

5. How was the birth of your child?
☐ Natural
☐ Scheduled cesarean
☐ Urgent cesarean
☐ I don't know

6. Did your child need to be sent to the neonatal ICU soon after being born?
☐ Yes
☐ No
☐ I don't know

7. Did your child need special medical treatment soon after being born?
☐ Yes
☐ No
☐ I don't know
If you answered yes, mark an X next to the special medical treatment:
☐ Needed treatment for jaundice ("yellowing of skin")
☐ Needed oxygen with intubation
☐ Needed oxygen without intubation
☐ Other. Which?...

From birth to 4 years of age

8. Did you child breastfeed?
☐ Yes
☐ No
☐ I don't know
If you answered yes, for how long?
☐ For less than six months
☐ For six months or longer
☐ I don't know

9. Did your child bottle feed with milk?
☐ Yes
☐ No
☐ I don't know
If you answered yes, for how long?
☐ For less than six months
☐ From six months to one year
☐ For more than one year
☐ I don't know

Was the milk heated?
☐ Yes
☐ No
☐ I don't know

How was the milk heated?
☐ In the microwave oven in a plastic bottle or plastic recipient
☐ In the microwave oven in a glass bottle or glass recipient
☐ On the stove, in a water bath using a pan
☐ On the stove directly in the pan

10. Did your child ever become sick from birth until 4 years of age?
☐ Yes
☐ No
☐ I don't know
If you answered yes, mark an X next to the illnesses your child had:
☐ Colds or the flu
☐ Pneumonia
☐ Respiratory infections (sinusitis, throat infection, pharyngitis, etc.)
☐ Ear infection (otitis)
☐ Other(s). Which?...

11. Did your child ever take medication from birth until 4 years of age?
☐ Yes
☐ No
☐ I don't know
If you answered yes, mark an X next to the medications your child took:
☐ Antibiotic (Amoxicillin, Clavulin, Amoxil, etc.)
☐ Analgesic/anti-thermal (Paracetamol, Dipyrone, Melhoral, etc)

☐ Anti-inflammatory (Nimesulide, Cataflam)
☐ Anti-allergic or medication for rhinitis
☐ Other(s). Which?..

12. Was your child ever hospitalized between birth and 4 years of age?
☐ Yes
☐ No
☐ I don't know
If you answered yes, mark an X next to the reason for hospitalization:
☐ Pneumonia
☐ Reflux
☐ Intoxication by medication
☐ Surgery
☐ Other(s). Which?...

13. Did your child ever have a high fever (equal to or higher than 38.5^{o} C) from birth until 4 years of age?
☐ Yes
☐ No
☐ I don't know

14. Did your child ever have asthma or bronchitis from birth until 4 years of age?
☐ Yes
☐ No
☐ I don't know
If you answered yes, did your child take medication? ☐ Yes
☐ No
☐ I don't know
If you answered yes, mark an X next to the medication(s) that your child took:
☐ Berotec
☐ Salbutamol or aerolin
☐ Prednisolone or predsim
☐ Acebrophylline
☐ Other(s). Which?...

15. Does your child have any relative with teeth that came in stained?
☐ Yes
☐ No
☐ I don't know
If you answered yes, what is the kinship with your child?................………………...........

16. Was your child born in Lavras?
☐ Yes
☐ No
☐ I don't know

17. Did your child live in Lavras from birth until 4 years of age?
☐ Yes
☐ No
☐ I don't know

Socioeconomic and demographic questionnaire

Child's name ____

Data of birth: _____/____/________ Gender: ( ) Male ( ) Female

Mother's name:____

Address:____

Neighborhood:_______________________________________ City:_________________________

Tel.__________________________

1- Mother's marital status:

☐ Single ☐ Married ☐ Divorced ☐ Other

2- The child's mother studied through to what year? _____________grade

3- The child's father studied through to what year? _____________grade

4- What is the monthly income of your family group? R$______________

5- How many people, including yourself, live on the monthly income of your family group?

☐ Two

☐ Three

☐ Four

☐ Five

☐ Six

☐ Seven or more. Write the number of people:_________

6- Number of children

☐ One ☐ Two ☐ Three ☐ Four ☐ Five ☐ More than five

7. How many older brothers and sisters does your child have?

☐ None

☐ One

☐ Two

☐ Three

☐ Four or more
