# Supplementary material for: Association between Molar Incisor Hypomineralization in Schoolchildren and Both Prenatal and Postnatal Factors: A Population-Based Study
Source: PLoS One. 2016 Jun 9;11(6):e0156332. doi: 10.1371/journal.pone.0156332 (PMC4900662; doi:10.1371/journal.pone.0156332)
Supplement: S2 File — (PDF) [file pone.0156332.s002.pdf]

QUESTIONÁRIO ÀS MÃES RELATIVO AO ESTUDO DOS PRIMEIROS MOLARES PERMANENTES DE CRIANÇAS

Obrigado por participar do nosso estudo!

Sua participação vai nos ajudar a obter um melhor entendimento do que faz com que alguns dentes nasçam malformados em algumas crianças. Algumas perguntas são sobre a saúde do seu filho, do nascimento aos quatro anos de idade e outras sobre a saúde da mãe durante a gravidez. Por favor, pense cuidadosamente e tente responder da melhor forma que puder.

Nome da criança:…………………………………………………...……………………..
Nome da mãe:……………………………………….............……………………...........

1. Qual foi o peso do seu(a) filho(a) quando nasceu?
☐ Menos de 1,5 kg
☐ De 1,5 kg a 2,49 kg
☐ Mais de 2,5 kg
☐ Não sei

2. Seu(a) filho(a) nasceu prematuro?
☐ Sim
☐ Não
☐ Não sei
Se você respondeu sim, com quantas semanas ele(a) nasceu?
☐ menos de 28 semanas
☐ De 28 a menos de 32 semanas
☐ De 32 a menos de 37
☐ Não sei

<u>3. Durante os dois últimos meses de gravidez, você teve:</u>
Proteinúria (perda de proteína pela urina)? ☐ Sim ☐ Não ☐ Não sei
Glicosúria (perda de glicose/açúcar pela urina)? ☐ Sim ☐ Não ☐ Não sei
Pressão alta? ☐ Sim ☐ Não ☐ Não sei
Pré-eclâmpsia? ☐ Sim ☐ Não ☐ Não sei
Outras doenças graves? ☐ Sim ☐ Não ☐ Não sei
Se você respondeu sim, quais foram as doenças graves?..
....

4. Durante os dois últimos meses de gravidez, você usou algum medicamento?
☐ Sim
☐ Não
☐ Não sei
Se você respondeu sim, marque com um X o medicamento usado:
☐ medicamento para pressão alta (ex.: Aldomet, etc)
☐ medicamento para “segurar” o bebê (ex.: Inibina, etc)
☐ Paracetamol
☐ Outro(s). Qual(is)?...……………………………………………..

5.Como foi o parto do seu(a) filho(a)?
☐ Natural
☐ Cesárea programada
☐ Cesárea de urgência
☐ Não sei

6. Logo após o nascimento, seu(a) filho(a) precisou ser encaminhado para a UTI neonatal?
☐ Sim
☐ Não
☐ Não sei

7. Logo após o nascimento, seu(a) filho(a) precisou de algum tratamento médico especial?
☐ Sim
☐ Não
☐ Não sei
Se você respondeu sim, marque com um X o tratamento médico especial:
☐ Precisou de tratamento para icterícia (“amarelão”)
☐ Precisou de oxigênio com entubação
☐ Precisou de oxigênio sem entubação
☐ Outro. Qual?...

Do nascimento até os 4 anos de idade

8. Seu(a) filho(a) mamou no peito?
☐ Sim
☐ Não
☐ Não sei
Se você respondeu sim, por quanto tempo?
☐ Por menos de 6 meses
☐ Por 6 meses ou mais tempo
☐ Não sei

9. Seu(a) filho(a) usou mamadeira com leite?
☐ Sim
☐ Não
☐ Não sei
Se você respondeu sim, responda:
Por quanto tempo? ☐ Por menos de 6 meses
☐ De 6 meses a 1 ano
☐ Por mais de 1 ano
☐ Não sei

O leite era aquecido? ☐ Sim
☐ Não
☐ Não sei

Como o leite era aquecido?
☐ No micro-ondas, dentro da mamadeira de plástico ou vasilha de plástico
☐ No micro-ondas dentro de mamadeira de vidro ou vasilha de vidro
☐ No fogão, em banho-maria, usando uma panela
☐ No fogão, direto na panela

10. Seu(a) filho(a) ficou doente do nascimento até os 4 anos de idade?
☐ Sim
☐ Não
☐ Não sei
Se você respondeu sim, marque com um X as doenças que seu(a) filho(a) teve:
☐ gripes ou resfriados
☐ Pneumonia
☐ Infecções respiratórias (sinusite, infeção de garganta, faringite, etc.)
☐ Infecção de ouvido (otite)
☐ Outra (s). Qual(is)?...

11. Seu(a) filho(a) usou medicamentos do nascimento até os 4 anos de idade?
☐ Sim
☐ Não
☐ Não sei
Se você respondeu sim, marque com um X os medicamentos que seu(a) filho(a) usou:
☐ Antibiótico (Amoxicilina, Clavulin, Amoxil, etc.)
☐ Analgésico/anti-térmico (Paracetamol, Dipirona, Melhoral, etc)
☐ Anti-inflamatórios (Nimesulida, Cataflan)

☐ Anti-alérgicos ou medicamentos para rinite
☐ Outro(s). Qual(is)?...

12. Seu(a) filho(a) foi internado em hospital do nascimento até os 4 anos de idade?
☐ Sim
☐ Não
☐ Não sei
Se você respondeu sim, marque com um X o motivo da internação:
☐ Pneumonia
☐ Refluxo
☐ Intoxicação com medicamento
☐ Cirurgia
☐ Outro(s). Qual(is)?..

13. Seu(a) filho(a) apresentou febre alta (igual ou maior do que 38,5^{o}C) do nascimento até os 4 anos de idade?
☐ Sim
☐ Não
☐ Não sei

14. Seu(a) filho(a) teve asma ou bronquite do nascimento até os 4 anos de idade?
☐ Sim
☐ Não
☐ Não sei
Se você respondeu sim, seu(a) filho(a) usou medicamentos? ☐ Sim
☐ Não
☐ Não sei
Se você respondeu sim, marque com um X o(s) medicamento (s) que seu(a) filho(a) usou:
☐ Berotec
☐ Sabutamol ou aerolin
☐ Prednisolona ou predsim
☐ Acebrofilina
☐ Outro(s). Qual(is)?..

15. Seu(a) filho(a) tem algum parente com dentes que nasceram manchados?
☐ Sim
☐ Não
☐ Não sei
Se você respondeu sim, qual o parentesco com a criança?................…………….............

16. Seu(a) filho(a) nasceu em Lavras?
☐ Sim
☐ Não
☐ Não sei

17. Seu(a) filho(a) morou em Lavras do nascimento até os 4 anos de idade?
☐ Sim
☐ Não
☐ Não sei

Questionário socioeconômico e demográfico

Nome da criança____

Data de nascimento: _____/____/________ Gênero: ( ) Masculino ( ) Feminino

Nome da Mãe:____

Endereço:____

Bairro:____ Cidade:__________________________

Tel.___________________________

1- Estado civil da mãe:

☐ Solteira; ☐ Casada; ☐ Divorciada; ☐ Outro

2- Até que série a mãe da criança estudou? _____________série

3- Até que série o pai da criança estudou? _____________série

4- Qual é a renda mensal de seu grupo familiar? R$______________

5- Quantas pessoas, incluindo você próprio, vivem da renda mensal do seu grupo familiar?

☐ Duas;

☐ Três;

☐ Quatro;

☐ Cinco;

☐ Seis

☐ Sete ou mais. Coloque o número de pessoas:_________

6- Número de filhos

☐ Um; ☐ Dois; ☐ Três; ☐ Quatro; ☐ Cinco; ☐ Mais de cinco

7. Seu(a) filho(a) tem quantos irmãos mais velhos do que ele?

☐ Nenhum

☐ Um

☐ Dois

☐ Três

☐ Quatro ou mais
